# Immunomagnetic Separation and Magnetic‐Susceptibility‐Based Quantification of AD Biomarkers Using Ultra‐Magnetic Iron Oxide Nanorod Agent Magfiniti^®^

**DOI:** 10.1002/alz.093456

**Published:** 2025-01-09

**Authors:** Yuancheng Li, Hui Mao, Jonathan Padelford, Hedi Ma

**Affiliations:** ^1^ 5M Biomed, LLC, Altanta, GA USA; ^2^ Emory University School of Medicine, Atlanta, GA USA; ^3^ 5M Biomed, LLC, Atlanta, GA USA

## Abstract

**Background:**

Enriching and detecting Alzheimer’s disease (AD) biomarkers in cerebral spinal fluid (CSF) or blood samples are increasingly applied in the AD diagnosis and monitoring of disease progression and treatment response. The accuracy of these processes is dependent on the sensitivity and specificity of capturing and quantifying AD biomarkers, e.g., Aβ40 and Aβ42, in the samples. This report introduces a new immunomagnetic separation based biomarker detection system, which employs AD‐biomarker‐targeting peptide functionalized ultra‐magnetic iron oxide nanorods Magfiniti^®^ to rapidly capture and separate AD biomarkers, coupled with measurement of change of the magnetic susceptibility upon capturing AD biomarkers.

**Method:**

The colloidal stability over 24 hours and magnetic separation efficiency of Magfiniti^®^ were determined using UV‐Vis spectroscopy. The capacity of Magfiniti^®^ in avoiding non‐specific protein adsorption, with Dynabeads^®^ as a control, was examined by incubating with human serum followed by quantifying adsorbed proteins. Synthetic γ‐AApeptides with proved targeting effect for Amyloid beta peptide 1‐40 (Aβ40) and 1‐42 (Aβ42) were conjugated to Magfiniti^®^ for detecting Aβ40 and Aβ42 spiked in human serum (100 to 2000 pg/mL). After a 30‐min incubation with serum samples, γ‐AApeptide‐conjugated Magfiniti^®^ with captured biomarkers were magnetically separated (10 min), washed (2X) with and re‐dispersed in PBS. Biomarker‐bound Magfiniti^®^ were then measured for the AC magnetic susceptibility (χ’) using a XacQuan II AC susceptometer, with blank Magfiniti^®^ with the same concentration measured as the baseline. The linear correlations between the difference of magnetic susceptibility (Δχ’) and Aβ concentrations were evaluated by the coefficient of determination (R^2^).

**Result:**

Magfiniti^®^ were stably dispersed in water, with >95% separated magnetically in 5 min. Magfiniti^®^ exhibited minimal adsorptions by serum proteins (∼5 µg/mg Fe) with the incubation time varying from 5 to 60 min, whereas proteins on Dynabeads^®^ ranged from 61.3 ± 12.7 to 223.3 ± 22.5 µg/mg Fe, as the incubation time increased. Excellent linear correlations (R^2^=0.9999) between Δχ’ and Aβ concentrations with standard deviations <1% were achieved, when Δχ’ were measured at the AC frequencies of 500, 1000, and 2000 Hz.

**Conclusion:**

The Magfiniti^®^‐based AD biomarker measurement may provide an alternative approach for liquid biopsy of AD using routinely collected blood samples.

